# New Treatment Options for Refractory/Resistant CMV Infection

**DOI:** 10.3389/ti.2023.11785

**Published:** 2023-10-12

**Authors:** Carla Simone Walti, Nina Khanna, Robin K. Avery, Ilkka Helanterä

**Affiliations:** ^1^ Division of Infectious Diseases and Hospital Epidemiology, Departments of Biomedicine and Clinical Research, University and University Hospital of Basel, Basel, Switzerland; ^2^ Division of Infectious Diseases, Department of Medicine, Johns Hopkins University, Baltimore, MD, United States; ^3^ Department of Transplantation and Liver Surgery, Helsinki University Hospital and University of Helsinki, Helsinki, Finland

**Keywords:** cytomegalovirus, antiviral resistance, antiviral therapy, letermovir, maribavir, virus-specific adoptive T cell therapy

## Abstract

Despite advances in monitoring and treatment, cytomegalovirus (CMV) infections remain one of the most common complications after solid organ transplantation (SOT). CMV infection may fail to respond to standard first- and second-line antiviral therapies with or without the presence of antiviral resistance to these therapies. This failure to respond after 14 days of appropriate treatment is referred to as “resistant/refractory CMV.” Limited data on refractory CMV without antiviral resistance are available. Reported rates of resistant CMV are up to 18% in SOT recipients treated for CMV. Therapeutic options for treating these infections are limited due to the toxicity of the agent used or transplant-related complications. This is often the challenge with conventional agents such as ganciclovir, foscarnet and cidofovir. Recent introduction of new CMV agents including maribavir and letermovir as well as the use of adoptive T cell therapy may improve the outcome of these difficult-to-treat infections in SOT recipients. In this expert review, we focus on new treatment options for resistant/refractory CMV infection and disease in SOT recipients, with an emphasis on maribavir, letermovir, and adoptive T cell therapy.

## Introduction

Following primary infection, cytomegalovirus (CMV) establishes lifelong latency in the human body. Seropositivity in adults ranges from 40% to 90% [1, 2]. After solid organ transplantation (SOT), reactivation of CMV is facilitated by drug-induced immunosuppression which is required to prevent and treat transplant rejection [1]. CMV remains one of the most common opportunistic infections in SOT and CMV disease affects overall around 5%–15% of patients despite preventive strategies [3–7]. Up to one-third of patients experience recurrent CMV [8], termed as repeated CMV after an interval without evidence of virus. For study purposes, “CMV infection” is defined as evidence of virus antigens or nucleic acid in any body specimen [9]. “CMV disease” is defined as additional presence of virus attributable signs or symptoms and includes CMV end-organ diseases and the “CMV syndrome”; The later is defined by detection of CMV in the blood together with at least two clinical findings including fever, malaise, leuko-, neutro- or thrombocytopenia, atypical lymphocytes or elevated liver enzymes [9].

The first line antiviral drug for CMV prevention and treatment is intravenous ganciclovir or its oral prodrug valganciclovir [10, 11]. This guanine analog requires phosphorylation by a viral kinase (UL97) for activation and inhibits the viral DNA polymerase (UL54) [1]. Neutropenia is a major toxicity occurring in 18%–47% [12]. Foscarnet and cidofovir are second-line treatments which also target the viral polymerase but their use is often limited by severe toxicities including nephrotoxicity in 14%–78% [8, 13–15]. Despite these well-established anti-CMV therapies, refractory and/or resistant (R/R) CMV provide a major challenge to clinicians [16].

CMV infection is clinically referred to as “refractory” if the viral load in the blood increases (>1 log_10_ compared to the maximum viral load in the first week) or persists after at least 2 weeks of appropriately dosed antiviral therapy [17]. Similarly, “refractory disease” is suspected if clinical signs or symptoms worsen or do not improve after 2 weeks of appropriate treatment [17]. A reduction in immunosuppression, an increase in the dose of ganciclovir, the addition of or a switch to second-line therapy, and resistance testing are then recommended [10, 11, 18]. In around one-third to half of refractory CMV cases, no drug-resistance can be detected [8, 13, 19]; suboptimal treatment responses may result from insufficient drug levels at site of infection.

“Resistant CMV” is defined as reduced susceptibility to one or more anti-CMV agents caused by viral gene mutation(s) [17]. In clinical practice, genotypic methods are used for diagnostics. Ganciclovir-resistant CMV occurs in around 1%–3% of SOT or 6%–18% of SOT recipients treated for CMV [4, 13, 18, 20–26], respectively, but may be more frequent in CMV seronegative recipients of organs from seropositive donors (D+/R− serostatus) [21, 25] and after lung transplantation [20]. Mutations in the UL97 gene are most frequent [1, 23]. UL54 mutations usually emerge upon extended pre-treatment and can confer cross-resistance with cidofovir and foscarnet [1]. Within the same gene, mutations in different codons are associated with varying levels of resistance [1]. Risk factors for drug-resistant CMV include D+/R− serostatus, lung transplant, high viral-loads, ongoing viral replication, prolonged antiviral exposure, subtherapeutic antiviral levels [4, 13], profound immunosuppression, and recurrent CMV infection [10, 18, 21, 23].

R/R CMV is further associated with complicated clinical courses including drug-toxicities, longer hospitalizations, and poor outcomes [17, 18, 27]; in a study of SOT recipients who were treated with foscarnet for ganciclovir-resistant or refractory CMV (*n* = 39; 0.66% of all SOT), 33% did not clear virus, 21% had recurrent CMV, and >50% had nephrotoxicities [13]. In lung and kidney transplants, R/R CMV was associated with increased frequencies of transplant dysfunction [18, 28]. Mortality seems also higher in resistant compared to non-resistant CMV in SOT; in a study that compared 39 ganciclovir-resistant cases with 109 ganciclovir-sensitive controls, mortality was 11% vs. 1% at 3 months, and 16% vs. 6% at 1 year after CMV diagnosis [18]. In summary, R/R CMV remains a major challenge and new effective and safe treatment options are needed.

**FIGURE 1:**
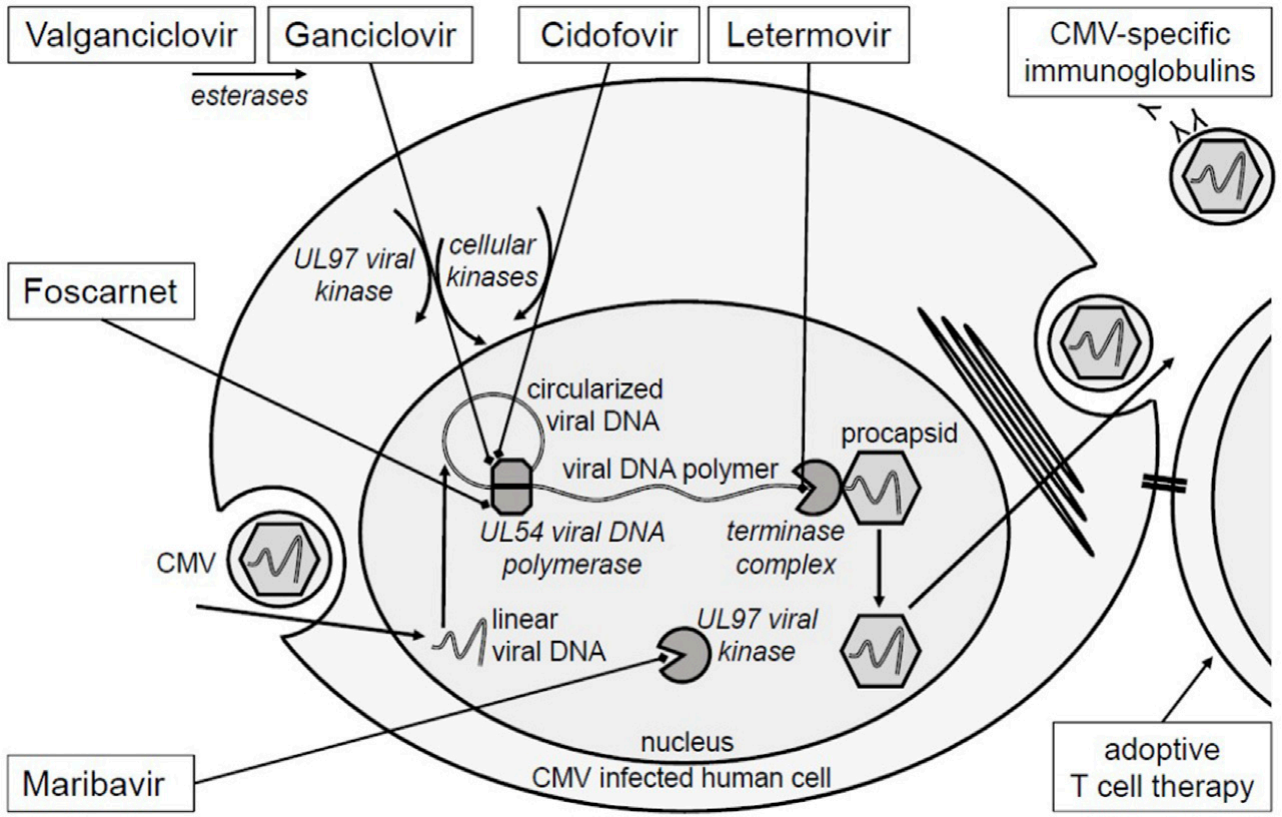
Mechanism of action of anti-CMV therapies. Ganciclovir and cidofovir are analogs of the phosphorylated nucleosides deoxyguanosine and deoxycytidine. Valganciclovir is an oral prodrug of ganciclovir. Ganciclovir requires phosphorylation by the *viral protein kinase* (UL97) for activation. Both, ganciclovir and cidofovir require phosphorylation by host *cellular phosphokinases* for activation**.** Both drugs competitively inhibit the *viral DNA polymerase* (UL54) at the desoxynucleotide triphosphate binding site. In contrast, foscarnet is an analog of pyrophosphate and inhibits UL54 at the pyrophosphate binding site. Maribavir has another target; by inhibition of the *viral protein kinase* (UL97), it inhibits phosphorylation of viral and host cellular proteins and consequently viral replication. Letermovir inhibits binding of the newly produced viral DNA polymers to the *viral terminase complex* [29, 30]. In this way it inhibits DNA cleaving and packaging into the viral procapsid. Mutations at the drug binding sites or in the activating viral kinases confer to resistances. CMV-specific adoptive T cells recognize CMV-infected cells via T cell receptor. Enzymes are displayed in italics.

In this review, we summarize and discuss the latest findings on maribavir, letermovir, and CMV-specific adoptive T cell therapies as treatment options for R/R CMV after SOT (summary in Table 1). Mode of action of established and new antivirals are shown in Figure 1.

**TABLE 1:** Advantages and limitations of new treatment options for refractory/resistant CMV in SOT.

	Mode of action	Advantages	Limitations
Maribavir	- Inhibition of viral UL97 kinase	- Well tolerated	- Dysgeusia in one-third of patients
		- Oral formulation	- No intravenous formulation
		- Efficacy demonstrated in a Phase 3 randomized controlled trial	- Reduced efficacy with high viral loads and in refractory CMV without resistance
		- Regulatory approval for this indication	- Poor penetration to CNS/retina
			- Drug-drug interactions
			- Recurrences after successful treatment
			- Resistances
Letermovir	- Inhibition of viral terminase complex	- Well tolerated	- No randomized controlled trials
		- Oral and intravenous formulation	- Approved only for prophylaxis
		- Combination therapy with ganciclovir possible	- Reduced efficacy with high viral loads
		- Possible option as secondary prophylaxis	- Relevant interaction with cyclosporine, sirolimus, tacrolimus
			- Recurrences after successful treatment
			- Resistances
CMV-specific adoptive T cell therapy	- Autologous or allogeneic *ex vivo* selected (and expanded) CMV-specific T cells to restore CMV-specific T cell immunity	- Mechanistic approach to restore immunity	- No randomized controlled trials
		- Reported to be safe	- Safety/efficacy await confirmation in Phase 3 trials
		- Alternative in drug resistant CMV	- Complex donor selection
		- Multi-virus specific commercial products under development	- Not widespread available
			- Time/cost intensive laboratory protocols
			- Expansion and function limited by immunosuppressive drugs

## New Treatment Options for R/R CMV

### Maribavir

#### History of the Drug Up Through the Phase 2 R/R Trial

Maribavir is an oral benzimidazole riboside antiviral which has been in development for many years, but only recently became available as therapy for R/R CMV. It inhibits viral UL97 kinase and thus interferes with multiple pathways including nuclear egress of CMV viral capsids. It has no significant renal, hematologic, or hepatic toxicity; its most common adverse effect is dysgeusia. Early trials for prophylaxis in stem cell transplant [31] and liver transplant recipients [32] failed to show efficacy, likely because the dose selected, 100 mg twice daily, was too low [33]. However, a case series of six patients with R/R CMV treated with compassionate use maribavir at doses of 400–800 mg twice daily showed striking responses in several patients [34]. This, and the toxicity of other agents available for R/R CMV, spurred the performance of a Phase 2 trial of 3 dosing regimens for maribavir (400, 800, and 1,200 mg twice daily) among SOT and HSCT recipients [19]. This study demonstrated clearance of CMV DNAemia at 6 weeks of therapy in 70%, 63%, and 68%, respectively, in this highly treatment-experienced population [19].

#### Phase 3 Trials

Subsequently, a multicenter Phase 3 trial of maribavir versus investigator-assigned therapy (IAT) was performed involving 352 SOT and HSCT recipients in a 2:1 randomization [8]. IAT, which could be ganciclovir, valganciclovir, foscarnet, cidofovir, or a combination of these, was chosen as the comparator because of patients’ varied treatment histories. The primary endpoint, confirmed CMV-DNA clearance at the end of week 8, was achieved by 55.7% in the maribavir arm vs. 23.9% in the IAT arm (*p* > 0.001). The key secondary endpoint, a composite of CMV-DNA clearance and symptom control at the end of week 8 maintained through week 16, was achieved by 18.7% vs. 10.3% (*p* = 0.01). Dysgeusia was the most frequent adverse effect in the maribavir group (37.2%); the maribavir group also had significantly less neutropenia than the val/ganciclovir group and less acute kidney injury than the foscarnet group [8]. These results led to the approval of maribavir by the US FDA in 2021 for treatment of post-transplant CMV infection/disease in patients age 12 and older, that is refractory (with or without genotypic resistance) to treatment with ganciclovir, valganciclovir, cidofovir or foscarnet, with a similar authorization by the EMA in 2022. A second Phase 3 randomized double-blinded trial (the AURORA trial, NCT02927067) compared maribavir to valganciclovir for treatment of asymptomatic CMV DNAemia in stem cell transplant recipients. At the time of this writing, full results have not yet been published, but topline results were announced by the study sponsor (Takeda) in December 2022. At week 8, which was the end of study treatment, 69.6% of patients treated with maribavir achieved CMV clearance vs. 77.4% for valganciclovir; this did not meet non-inferiority based on a prespecified margin of 7%. At week 16, 52.7% of patients treated with maribavir achieved maintenance of viremia clearance and symptom control vs. 48.5% for valganciclovir. Similar post-treatment maintenance effect was observed at week 12 (59.3% vs. 57.3%) and week 20 (43.2% vs. 42.3%) time points. Maribavir’s safety profile was confirmed, particularly with regards to neutropenia (21.2% vs. 63.5% for valganciclovir). Despite not meeting the prespecified noninferiority margin, this study demonstrated that maribavir has potential utility for treatment of non-refractory CMV DNAemia, with a lower risk of hematologic toxicity than valganciclovir.

#### Questions About Optimal Use

While the approval of maribavir for R/R CMV was long-awaited, questions about optimal use remain. In the Phase 3 R/R CMV trial, subgroup analyses showed that the proportion achieving the primary endpoint was higher when maribavir was initiated at a viral load of <9100 IU/mL than at higher viral loads (62.1% vs. 43.9%), and was higher with documented genotypic resistance vs. refractory CMV without resistance (62.8% vs. 43.8%) [8]. Some experts have proposed that R/R CMV with high viral load might most effectively be treated with an agent such as foscarnet initially, then switch over to maribavir at a lower viral load, to minimize foscarnet toxicity and to maximize the efficacy of maribavir [35]. Another issue, as with all therapies for R/R CMV, is the risk for recurrences. While maribavir achieved the key secondary endpoint significantly more often than IAT, the numbers in both groups were relatively low (who maintained CMV clearance and symptom control out to week 16 after completion of therapy at week [8]). Of note, the Phase 3 R/R maribavir trial [8] did not permit secondary prophylaxis after the defined 8 weeks treatment period, whereas the Phase 2 R/R maribavir study had allowed continuation of maribavir out to 24 weeks [19]. Whether secondary prophylaxis would be of benefit (in terms of decreasing recurrences after CMV DNAemia clearance), and whether that would be offset by potential increases in maribavir resistance, has yet to be studied, but will be important to assess. Although the evidence supporting the use of secondary prophylaxis is mostly lacking, many centers use secondary prophylaxis, and current guidelines recommend considering secondary prophylaxis in high-risk scenarios [10]. Combination therapy with maribavir is also a promising frontier that is yet to be explored. Chou et al. demonstrated that the maribavir/ganciclovir combination is antagonistic, and additive for maribavir + foscarnet or cidofovir or letermovir, but synergistic for maribavir + rapamycin (sirolimus) [36]. The use of an mTOR inhibitor-based immunosuppressive regimen is another strategy in prevention or management of R/R CMV particularly in organ transplant recipients [37]. The maribavir + mTOR inhibitor combination deserves further study.

#### Resistance

Perhaps the most important questions regarding its future utility relate to the risk for development of resistance to maribavir. An impressive body of work by Chou has addressed this issue for nearly 20 years, now utilizing updated sequencing technology [38]. Chou et al. analyzed resistance mutations from the Phase 2 maribavir trials, and found known UL97 maribavir resistance mutations after 46–166 days of maribavir therapy (T409M or H411Y) in 17 of 23 who had had CMV recurrences while on maribavir [39]. Moreover, they identified the mutation UL97 C480F in six patients, which confers high-level maribavir resistance and low-level ganciclovir resistance [39]. A recent real-world case series described maribavir resistance in 4 of 13 patients treated for R/R CMV (with H411Y in 2, T409M in 1, and C480F in 1) [40]. Another report described two patients refractory to maribavir, one with H411Y and one without known maribavir resistance mutations [41].

#### Conclusion

Maribavir has far less toxicity than other agents for R/R CMV, and is a major advance in treatment of this entity. However, we still have much to learn about optimizing its use and preventing recurrences and resistance.

### Letermovir

#### Background and Mechanism of Action

Letermovir is a 3,4-dihydroquinazoline derivative and is an inhibitor of the viral terminase complex, mainly at the pUL56 subunit. Terminase inhibition leads to compromised viral replication by inhibiting the cleavage of genome particles to units of proper length and accumulation of immature viral DNA [29]. Based on the mechanism of action, letermovir is selectively active only against CMV, and mechanism-derived adverse effects are unlikely. Letermovir was approved in 2017 for prophylactic use in adult CMV-seropositive allogeneic hematopoietic stem cell transplant (HCT) recipients, where it has shown good efficacy in the placebo-controlled phase III trial [42] and as of 6 June 2023, the US FDA approved letermovir for the new indication of CMV prophylaxis in D+/R− kidney transplant recipients, based on the results of the Phase 3 trial [43]. No statistically significant differences were seen in the frequency or severity of any adverse events between letermovir and placebo, although gastrointestinal adverse events (such as nausea) were slightly more common in the letermovir group. It is available in both peroral (PO) and intravenous (IV) formulations. The standard dose is 480 mg daily (IV/PO) when used as prophylaxis. However, due to interaction via the hepatic drug transporter organic-anion-transporting polypeptide (OATP), cyclosporine increases bioavailability of letermovir, and dose reduction to 240 mg daily is recommended [43].

#### Letermovir Prophylaxis Among SOT Recipients

In the phase 3 trial, 601 CMV D+/R− adult kidney transplant recipients were randomized to receive prophylaxis with either valganciclovir or letermovir 480 mg once daily (240 mg if used with cyclosporine) until week 28 after transplantation. Primary efficacy endpoint of the study was met, as letermovir was non-inferior to valganciclovir in preventing CMV disease (frequency 10.4% in the letermovir vs. 11.8% in the valganciclovir group). Importantly, letermovir resulted in lower toxicity compared to valganciclovir, especially lower rate of leukopenia (11.3% vs. 37%) or neutropenia (2.7% vs. 16.5%), and lower rate of drug discontinuation due to adverse events (4.1% vs. 13.5%) [43]. The study results are very convincing for the good efficacy of letermovir also in the SOT setting, when used as prophylaxis, and have recently led to the expanded indication mentioned above, by the US FDA.

#### Letermovir for Treatment of CMV Infections, Background

Larger industry-driven studies have all addressed the use of letermovir only as CMV prophylaxis, but due to lack of suitable alternatives for treating resistant CMV infections until recently, there has similarly been interest on using letermovir for treatment of CMV infections. However, as the drug does not block viral DNA synthesis, but inhibits events later in the viral cycle, some concerns have been raised about the potential to promote resistant viral strains, especially when used in case of high-level viremia. Indeed, several mutations in the pUL56 subunit of the terminase complex have been described after exposure to letermovir, potentially causing resistance to the antiviral action of the drug [44]. Interestingly however, in the phase 3 kidney transplant trial, no letermovir resistance-associated substitutions/mutations were detected in the letermovir arm, in comparison to nine patients in the valganciclovir arm, who developed ganciclovir resistance-associated mutations [45].

#### Letermovir for Treatment of CMV Infections, Real-World Experience

Table 2 briefly summarizes published case series of studies using letermovir as treatment of CMV infections. Most common dose has been 480 mg once daily PO, but also higher doses (up to 960 mg daily) have been used. In these studies, 76% of the cases with CMV infection treated with letermovir resulted in either viral clearance or decrease to viremia <200 IU/mL, and treatment failure was seen in 24% of cases. Although letermovir was mainly effective and resulted in lowering of viremia or viremia clearance, recurrent infections were common. In the multicenter retrospective study by [46], viral suppression was more likely when letermovir was started at a viral load of <1000 IU/mL. Therefore, another option worth considering would be to treat the viral load to low levels with another agent such as foscarnet, and then switch to letermovir to maximize the chance of clearance and minimize foscarnet toxicity.

**TABLE 2:** Studies or case series reporting the use of letermovir (LTV) for treatment of refractory/resistant CMV infection, or after failure to tolerate first-line treatment.

Author/journal/year	Type of SOT and number of patients	Reason for LTV treatment	Dose of LTV	Outcomes
Linder et al. [46]	27 SOT (13 lung, 6 kidney, 2 heart, 1 liver, 5 other)	Intolerance to other antivirals (77%), resistance concerns (33%)	480 mg OD: 87%	Good virologic outcomes if viral load <1,000 IU/mL at starting LTV; if > 1,000 IU/mL at starting, only approx. 40% reached DNAemia <1,000 IU/mL
Transplant Infect Dis 2021	In addition, 21 HCT included		720 mg OD: 13% (titrated up to 960 mg in two patients)	
			Oral: 89%	
			Intravenous: 11%	
Veit et al. [47]	28 SOT (all lung)	Refractory infection (57%), confirmed antiviral resistance (43%)	480 or 240 mg OD (based on tacrolimus or cyclosporine use)	Decrease in viral load within median 17 days and subsequent clearance in 82%; treatment failure in 18%
Am J Transplant 2021				
Schubert et al. [48]	5 SOT (3 kidney, 2 heart)	refractory infection (11%), intolerance to other antivirals (67%), confirmed resistance (22%)	480 or 240 mg OD (based on tacrolimus or cyclosporine use)	Decrease in viral load to <200 IU/mL within median 23 days seen in 78%
Eur J Clin Microbiol Infect Dis 2021	In addition, two HSCT and two other immunosuppressed patients included			
Ortiz et al. [49]	4 SOT (3 SPK, 1 kidney)	Intolerance to (val)ganciclovir (50%), confirmed antiviral resistance (50%)	480 or 240 mg OD (based on tacrolimus or cyclosporine use)	Viral clearance reached in 75%, and decrease in viral load to <200 IU/mL in 25%, after 4–9 weeks of treatment
Clin Transplant 2022				
Phoompoung et al. [50]	4 SOT (lung), in addition one HSCT included	Refractory infection (50%), intolerance to other antivirals (25%), confirmed antiviral resistance (25%)	480 or 240 mg OD (based on tacrolimus or cyclosporine use)	Decrease in viral load to <200 IU/mL within 3–6 weeks in 75%, treatment failure in 25%
Transplantation 2020				
Turner et al. [51]	4 SOT (2 lung, 2 heart)	confirmed antiviral resistance	720 mg OD, dose titrated up to 960 mg in one patient	All showed clinical improvement, virological treatment failure in 75%
Antimicrob Agents Chemother 2019	CMV retinitis in all			
Aryal et al. [52]	2 SOT (lung, heart)	confirmed antiviral resistance	480 or 240 mg OD (based on tacrolimus or cyclosporine use)	viremia clearance in 50%, treatment failure in 50%
Transplant Infect Dis 2019	In addition, 7 patient included with LTV prophylaxis			
Boignard et al. [53]	2 SOT (heart)	intolerance to other antiviral (50%), confirmed resistance (50%)	480 mg OD	Viremia clearance in 50%, treatment failure in 50%
Antiviral Ther 2022				

Significant interaction with tacrolimus was noted, and tacrolimus dose needed to be adjusted (reduced significantly) in many cases. Letermovir is a moderate inhibitor of CYP3A *in vivo* [54], and therefore leads to increase in tacrolimus and cyclosporine (and sirolimus) concentrations. In phase 1 studies, coadminstration of letermovir with tacrolimus or cyclosporine resulted in 2.4- and 1.7-fold increases in area under the plasma concentration-time curves, and 1.6- and 1.1-fold increases in maximum plasma concentrations, respectively [55].

The use of letermovir as an antiviral agent in preemptive therapy after solid-organ transplantation has been so far addressed in only one early proof-of-concept phase 2a study, in which antiviral efficacy was shown despite using much lower doses than the current recommendation (only 80 mg/day) [56]. Some more experience of successful use of letermovir as preemptive therapy after HSCT has been described [57].

Combination therapy with letermovir and (val)ganciclovir or CMV IvIG has also been reported. In the largest study reporting combination therapy so far, eight kidney or kidney-pancreas recipients with persisting low-level viremia despite >90 days of valganciclovir were treated with valganciclovir 900 mg twice daily together with letermovir 480 mg once daily. In this study, the use of adjunctive letermovir did not result in viral clearance, and median viral load did not change during 12 weeks of follow-up.

Suggested or confirmed genotypic resistance to letermovir was described in some of the case series, and in addition in case reports. In total at least seven genotypically resistant cases have been published to date after solid-organ transplantation, with mutations seen in UL56 gene [46, 47, 51, 58]. Similarly, mutations in UL56 have been described in patients who received letermovir prophylaxis after HSCT [59]. However, the vast majority of CMV infections treated with letermovir have not resulted in resistance concerns.

#### Future Directions

Based on the published experience so far and our own clinical experience, letermovir can be considered for treatment of R/R CMV infections. Favorable results will more likely be reached if treatment is initiated at low-level viremia, but recurrence and development of resistance are remaining concerns. In cases of poor tolerance to valganciclovir due to leukopenia or neutropenia, the potential to use letermovir as secondary prophylaxis after clearance of viremia could be further explored. However, some concerns about breakthrough infections and emergence of letermovir resistance have been raised in small case series [52, 60].

### CMV-Specific Adoptive T Cell Therapy

#### Rational for CMV-Specific Adoptive T Cell Therapy

T cell immunity is essential for CMV control [61, 62]. In SOT recipients, T cell immunity is weakened by immunosuppressive drugs, making direct restoration of immunity by infusion of CMV-specific T cells (“adoptive” T cell therapy) attractive [63].

To date, most clinical data on CMV-specific T cell therapies derive from phase 1/2 studies in allogeneic HCT recipients in which cells were infused for CMV-prophylaxis or treatment of R/R CMV [64]. Different protocols for T cell generation and application including intrathecal administration [65] were demonstrated to be safe and treatment for R/R CMV was successful in around 70% [64, 66]. Despite these promising data, the safety and efficacy still need to be confirmed in phase 3 studies. Additionally, there is very little data on SOT recipients.

#### T Cell Donors

Traditionally, CMV-specific T cells were harvested from the HCT donor. This limited the treatment to HCT recipients with CMV seropositive donors. More recently, peripheral blood cells from only partially HLA-matched CMV seropositive third-party donors were also successfully used [67]. This enabled therapy also in SOT recipients. Third-party cells were either collected prior and stored for “off-the-shelf” use [67] or collected upon request from pre-screened individuals in donor registers [68, 69]. Despite concerns about limited proliferative capacity due to continued immunosuppression, studies have shown successful expansion of autologous virus-specific T cells [70–73].

#### Preparation and Availability

*Ex vivo* steps are required to exclusively select CMV-specific T cells from the original donor product [64]. Complex and time intensive laboratory expansion protocols of minimum 10 days but up to 30 days are used to obtain high numbers of specific T cells [72, 74]. Alternatively, CMV-specific donor-derived white blood cells are directly isolated *ex vivo* using immunomagnetic methods (e.g., direct sorting using peptide-HLA multimers, cytokine-capture system or based on T cell activation molecules) [75–77].

Adoptive T cell therapies are still mainly restricted to specialized academic centers and few commercial companies due to the complexity of donor search and selection and the requirement of “good manufacturing practice”-accredited laboratories to prepare the cells *in vitro*. However, in recent years, increasing number of centers were able to offer “off-the-shelf” products to their patients as part of multicentric trials (e.g., NCT04390113 and [67]).

#### Safety

Virus-specific adoptive T cell therapies are generally reported to be safe. For allogeneic products, graft-versus host disease is a potential concern despite viral-specificity of most cells and was reported in around 5%–16% [64]. Independent of cell source, cytokine release syndrome and graft failure due to T cell mediated inflammation may occur but have rarely been reported [73, 78]. An open issue is the co-administration of immunosuppressive drugs, which affects the expansion and function of T cells *in vivo* after infusion into the patient. The optimal timing and composition of immunosuppression at the time of virus-specific T cell infusion remains to be determined.

#### CMV-Specific Adoptive T Cell Therapy in SOT

At this time, data from 19 SOT recipients treated with CMV-specific T cells have been reported, including one pediatric patient of 16 years of age, 11 lung, 6 kidney, 1 heart, and 1 liver transplant recipient (Table 3, including one unpublished case from our institution) [69–73, 79]. All recipients were treated for R/R CMV infection (*n* = 5) or disease (present or recent, *n* = 14). Anti-CMV drug resistance was reported in 12 cases. All protocols collected T cells from peripheral blood and most used *ex vivo* expanded cells. At our institution, we have successfully used the cytokine-capture system to isolate CMV-specific T cells.

**TABLE 3:** Case reports and one case series reporting the use of CMV-specific adoptive T cell therapy in SOT.

Author/journal/year	Type of SOT and number of patients	Reason for treatment with CMV-specific T cells	T cell donor/Strategy	Outcomes
Smith et al. [72]	13 SOT (4 kidney, 8 lung, 1 heart)	Recurrent, refractory and/or resistant CMV infection/disease or any CMV infection/disease with drug intolerance	Autologous	Objective improvement of symptoms, including reduction/resolution of DNAemia in 85% (11/13). Adverse events were of grade 1 (nausea, malaise, fatigue, altered taste sensation) and 2 (fatigue, halitosis, microangipathic hemolytic anemia)
Clinical Infectious Diseases 2019			*Ex vivo* expanded	
			1–6 doses; 22.2–224 × 10^6^ T cells/dose. 8/13 with concomitant antiviral therapy after infusion	
Brestrich et al. [73]	1 SOT (lung)	Recurrent, refractory CMV-pneumonia on mechanical ventilation	Autologous	Virologic and clinical response after 1st dose. Recurrent pulmonary CMV disease 6 weeks later. Died from CMV-negative graft failure
Am J Transplant 2009			*Ex vivo* expanded	
			One dose as treatment (1 × 10^7^ cells/m^2^), 2nd dose as secondary prophylaxis	
Holmes-Liew et al. [71]	1 SOT (lung)	Recurrent, resistant CMV infection after resolved CMV disease (hepatitis, pancytopenia)	Autologous	CMV PCR undetectable at time of infusions and for 16 months following infusion
Clin Transl Immunology 2015			*Ex vivo* expanded	
			Four doses (3 × 10^7^ T cells/dose)	
Pierucci et al. [70]	1 SOT (lung)	Recurrent, resistant CMV infection with intolerance to cidofovir	Autologous	CMV titer reduction but no clearance. Died from unrelated fungal infection
J Heart Lung Transplant 2016			*Ex vivo* expanded	
			Three doses (1.9–2.2 × 10^7^ T cells/dose)	
Macesic et al. [69]	1 SOT (kidney)	Recurrent, resistant CMV disease (glomerular thrombotic microangiopathy)	Allogeneic (3/6 HLA matched third-party donor from a donor bank)	Virologic and clinical response but remained dialysis dependent. Mild fever following infusion
Am J Transplant 2015			*Ex vivo* expanded	
			One dose (1.6 × 10^7^ T cells/m^2^). Concomitant artesunate	
Miele et al. [79]	1 SOT (liver)	Recurrent, refractory CMV disease (leukopenia, thrombocytopenia, interstitial pneumonia)	Allogeneic (5/6 HLA matched mother)	Virologic and clinical response (leukopenia resolved). No CMV relapse in the following 10 years
Microorganisms 2021			*Ex vivo* expanded	
			Two doses (1st dose with 1 × 10^6^ cells/kg)	
Stuehler C., Khanna N. et al. University Hospital of Basel, Switzerland (unpublished data)	1 SOT (kidney)	Recurrent, refractory CMV infection after CMV disease (leukopenia, pneumonia)	Allogeneic (6/6 HLA matched daughter)	Clinical response (leukopenia resolved). Partial virologic response with ongoing low-level replication under valganciclovir
			Immune magnetic sorting using cytokine capture assay	
			One dose (3.5 × 10^4^ cells/kg)	

Sixteen patients received autologous T cells and interestingly, it was possible to harvest CMV-specific T cells from patients with CMV D−/R− and D+/R− serostatus at time of transplantation [72]. In one patient, the immunosuppressive treatment was reduced specifically for cell harvesting, and the authors recommended this measure 2–3 weeks prior to cell collection [70].

Three patients received fully or partially HLA-matched third-party allogeneic T cells; our patient received the cells from his HLA-matched daughter, the pediatric patient received cells from his mother who was not the SOT donor [79], and another patient received cells from a third-party donor who was selected from a donor registry [69].

One to six doses of CMV-specific T cells were infused per patient with single doses between 0.24 × 10^7^ and 3 × 10^7^ cells. After infusion, some trials observed rapid *in vivo* expansion of CMV-specific T cells with simultaneous drop in viral load [73], however, other protocols could not observe these dynamics [70].

Infusions were generally well tolerated. Smith et al observed in their case series only grade 1 and 2 adverse events with potential association to the T cell infusion [72]. No graft-versus-host disease was observed with the allogeneic products, however, one patient had a mild fever following infusion which was potentially associated with cytokine release [69]. Of note, in the very first reported case, a lung transplant recipient with a drug-resistant CMV pneumonia on mechanical ventilation initially responded clinically and virologically after a first infusion of autologous CMV-specific T cells, could be discharged, and received a second infusion for prophylaxis, however, he subsequently died few weeks later from CMV-negative graft failure and it was not possible to fully exclude an association with the T cell therapy [73]. No changes in graft status were observed in the other cases.

As cases were not controlled and concomitant antiviral-drug regimen were often present, larger and controlled studies are necessary to estimate and prove treatment efficacy (e.g., as for BK virus in kidney transplantation, NCT04605484).

In summary, CMV-specific adoptive T cell therapy is an appealing option for R/R CMV in SOT. However, safety and efficacy need to be confirmed in controlled trials. Additional data is needed to identify the best protocols in terms of T cell generation and optimal time point of application and the influence of different immunosuppressive therapies on treatment efficacy should be investigated. At this point, we recommend that CMV-specific T cell therapies should be preferentially offered within clinical trials in order to close the knowledge gaps.

### Other Options

Other options for treatment of R/R CMV in SOT have been discussed in the latest guidelines [10, 11]; brincidofovir, an oral conjugated form of cidofovir, is US FDA approved for smallpox as bioterrorism agent but no longer available [80] after it failed as prophylaxis for CMV in a phase 3 trial in HCT [81]. Use of leflunomide [82] or artesunate, both with *in vitro* efficacy against CMV remains anecdotal [83, 84]. And although 31% of respondents in a recent survey among mainly European SOT centers reported that they add CMV-specific immunoglobulins to the antiviral therapy for ganciclovir-resistant CMV [16], this approach is controversial. The current guidelines state that randomized trials are needed to adequately investigate the role of CMV-specific immunoglobulins [10, 11].

Reduction of immunosuppressive drug doses to lowest doses compatible with graft survival remains fundamental in CMV treatment. However, type of immunosuppression might also play a role; data of a recent meta-analysis suggested that compared to calcineurin inhibitors alone the addition of everolimus may be associated with lower risk for CMV infection and similar trends were observed with other mTOR inhibitors [37]. In contrast, mycophenolate mofetil might increase risk for CMV disease [85] and therefore, many clinicians hold the drug during R/R CMV episodes.

## Conclusion

While R/R CMV remains an important complication in SOT, new therapeutic options became available in the recent years (Table 1).

Best evidence on efficacy and safety is available for maribavir and we therefore recommend maribavir as first-line treatment for R/R CMV in SOT. However, although maribavir was superior to standard therapies for R/R CMV, many patients did not achieve sustained viral clearance and symptom control. Especially patients with high initial viral loads and patients without genotypic resistance might be at risk for suboptimal responses, and, because of poor drug penetration, patients with CMV encephalitis and retinitis were completely excluded from the pivotal trial. Additionally, maribavir resistance and drug-drug interactions might become more relevant with broader use. This underlines the need for alternative strategies and still legitimates use of the conventional second-line drugs, foscarnet and cidofovir, depending on the individual patient situation.

More studies are needed to define the role of letermovir in R/R CMV; its best use may be in secondary prophylaxis. However, small case series reported a favorable response to treatment of R/R CMV infections.

Similarly, few data are currently available on safety and efficacy of CMV-specific T cell therapy in SOT. Until further data are available, we recommend treatment in clinical trials.

Authors’ institutional guidelines and personal insights are shown in Table 4.

**TABLE 4:** Refractory/resistant CMV treatment strategies at Helsinki University Hospital, Johns Hopkins University, and University Hospital of Basel.

	Helsinki University Hospital, Finland	Johns Hopkins University, United States	University Hospital of Basel, Switzerland
Testing	Genotypic test for drug resistance only in selected cases with risk factors and failure to respond despite to 21 days of adequately dosed therapy	Genotypic test for drug resistance in patients without response despite 14 days of adequately dosed therapy	Genotypic test for drug resistance in patients without response despite 14 days of adequately dosed therapy
Current strategy to treat refractory/resistant CMV	Letermovir has been used in selected cases with success. Generally try to avoid foscarnet due to nephrotoxicity. Until recently, Maribavir has not been available	Maribavir is now considered first-line therapy for R/R CMV infections at many centers. However, if the starting CMV viral load is extremely high, some clinicians may try to decrease the CMV viral load with another agent such as foscarnet first, then switch to maribavir after a drop in viral load and before significant toxicity has occurred	Foscarnet. In some cases addition of CMV specific immunoglobulins. Early discussion of treatment with adoptive CMV-specific adoptive T cell therapy from third party donor (ongoing phase 1/2 study). Maribavir was not readily available until to date
Planned adaption to the strategy	Maribavir as first line therapy in r/r CMV infection and disease	Maribavir as first line therapy in r/r CMV infection and disease. In future also hope to use maribavir for those with CMV recurrences and prior neutropenia during CMV treatment	Maribavir as first line therapy in r/r CMV infection and disease
Personal view	Most of the r/r CMV infections can be successfully treated with (val)ganciclovir together with mild reduction in immunosuppression and long enough courses of treatment, but leukopenia during long treatment is a problem	Collaboration between transplant teams and transplant infectious disease specialists essential; reduction of immunosuppression; Ig supplementation for hypogammaglobulinemic patients. Need further study of benefits/risks of secondary prophylaxis	Close management within interdisciplinary teams including transplant care team and infectious disease specialists recommended. We generally omit cidofovir due to nephrotoxicity
